# Supplementary material for: Resumption of Cyclic Ovarian Activity by Herbal Preparation AyuFertin in Bulgarian Murrah Buffaloes at Early Postpartum
Source: Animals (Basel). 2021 Feb 6;11(2):420. doi: 10.3390/ani11020420 (PMC7915216; doi:10.3390/ani11020420)
Supplement: Supplementary file 1 [file animals-11-00420-s001.pdf]

Supplementary material: Figures S1-S3 for the article Ilieva et al. "**Resumption of cyclic ovarian activity by the natural precursors of prostaglandins from AyuFertin in Bulgarian Murrah buffaloes at early postpartum**"

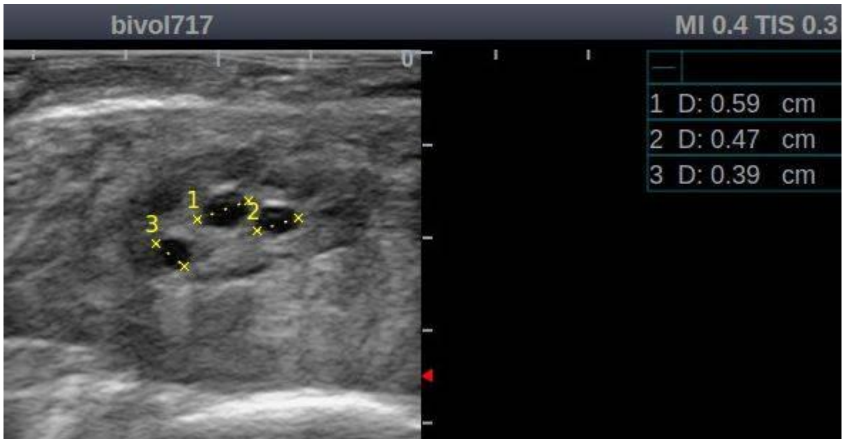

Figure S1. Small follicles in the ovaries of Bulgarian Murrah buffalo at postpartum day 20 (before treatment with AyuFertin).

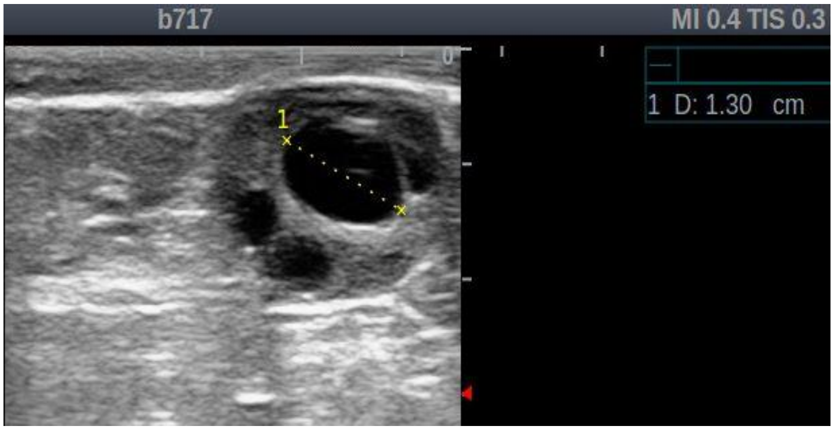

Figure S2. Large and medium follicles in the ovaries of Bulgarian Murrah buffalo at postpartum day 24 (after the 1st treatment with AyuFertin).

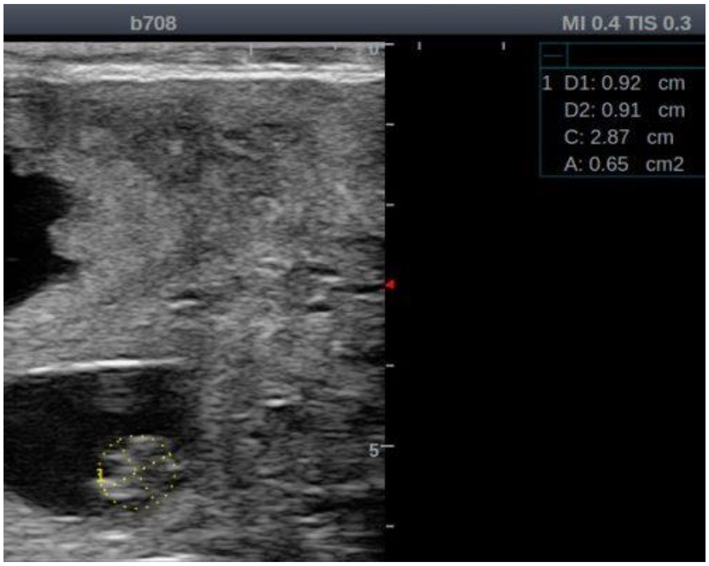

Figure S3. Pregnancy in experimental Bulgarian Murrah buffalo at day postpartum 70.
